# Impact of a Mediterranean diet, physical activity, body composition, and insulin delivery methods on metabolic control in children with type 1 diabetes

**DOI:** 10.3389/fnut.2023.1338601

**Published:** 2024-01-05

**Authors:** Yeray Nóvoa-Medina, Alicia Pérez-Lemes, Nerea Suárez-Ramírez, Marta Barreiro-Bautista, Himar Fabelo, Sara López-López, Sofia Quinteiro, Angela Domínguez, Marta León, María A. González, Elisabeth Caballero, Ana M. Wägner

**Affiliations:** ^1^Pediatric Endocrinology Unit, Complejo Hospitalario Universitario Insular Materno Infantil de Las Palmas de Gran Canaria, Las Palmas de Gran Canaria, Canary Islands, Spain; ^2^Asociación Canaria para la Investigación Pediátrica (ACIP Canarias), Las Palmas de Gran Canaria, Canary Islands, Spain; ^3^Instituto Universitario de Investigación Biomédica y Sanitaria (IUIBS), Las Palmas de Gran Canaria University, Las Palmas de Gran Canaria, Canary Islands, Spain; ^4^Faculty of Medicine, Las Palmas de Gran Canaria University, Las Palmas de Gran Canaria, Canary Islands, Spain; ^5^Complejo Hospitalario Universitario Insular—Materno Infantil, Fundación Canaria Instituto de Investigación Sanitaria de Canarias, Las Palmas de Gran Canaria, Spain; ^6^Research Institute for Applied Microelectronics, Universidad de Las Palmas de Gran Canaria, Las Palmas de Gran Canaria, Spain; ^7^Endocrinology and Metabolism Unit, Complejo Hospitalario Universitario Insular Materno Infantil de Las Palmas de Gran Canaria, Las Palmas de Gran Canaria, Canary Islands, Spain

**Keywords:** type 1 diabetes, children, HbA1c, nonpharmacological, diet, physical activity, body composition

## Abstract

**Aims:**

To evaluate the synergistic impact of diet, lifestyle and technology on glycemic control in children with type 1 diabetes (T1D).

**Methods:**

This cross-sectional study included 112 randomly selected patients with T1D from Gran Canaria (median age 12 years; 51.8% female). The study collected data on height, weight, body composition (bioimpedance), age, disease duration, and method of insulin delivery. Physical activity was evaluated using the Krece questionnaire and an accelerometer (GENEActiv). Adherence to the Mediterranean diet was assessed using the KIDMED Quick Nutrition Test. Glycemic control was evaluated using HbA1c and the percentage of time in range. SPSS version 21 and RStudio were used for statistical analysis of the data. Stepwise linear regression analysis (backwards) was used to identify factors independently associated with metabolic control.

**Results:**

Insulin pump use, age and adherence to the Mediterranean diet were found to be significantly and independently associated with better glycemic control, whereas years with T1D was associated with worse HbA1c values. No relationship was found between body composition and physical activity measured by accelerometry or questionnaire.

**Conclusion:**

Adherence to the Mediterranean diet, insulin delivery methods, age, and number of years with T1D are important factors to consider in the management of T1D in children.

## 1 Introduction

Since the introduction of insulin therapy in 1922 (1), the life of people with diabetes has greatly improved. After the results of the Diabetes Control and Complications Trial (DCCT) were published (2, 3), demonstrating the impact of intensive therapy on the development of vascular complications, more stringent metabolic control has become the standard of care for type 1 diabetes (T1D) patients. Recommended control targets have been decreasing over the years, with current advances such as continuous glucose monitoring and integrated insulin pumps helping to diminish the risk of hypoglycemia (4). International guidelines currently recommend an HbA1c level less than 7% or more than 70% of the time in range (interstitial glucose concentration between 70 and 180 mg/dL) as the goal of treatment for pediatric patients with T1D (4, 5), whereas national guidelines such as the National Institute for Health and Care Excellence (NICE) from the United Kingdom recommend even lower targets (6.5%) (6).

Alongside insulin therapy, nutrition and physical activity are important pillars of T1D management. These factors favor overall health in youth with T1D and are, therefore, routinely included in the guidelines of the American Diabetes Association (ADA) (4) and the International Society for Pediatric and Adolescent Medicine (ISPAD) (5). High rates of concurrent overweight and obesity are reported in youth with T1D, and some studies show up to 9% more body fat in these individuals than in children without T1D (7). Additionally, excessive body fat has been shown to increase the risk of cardiovascular disease in children and adults (8) and to have a negative impact on glycemic management (9).

It is increasingly recognized that strategies that emphasize activity and a healthy diet are needed, as studies have shown that children who are more active (10) and who adhere to the Mediterranean diet, which focuses on vegetables, fruits, whole grains, beans, nuts, and legumes with some lean proteins from fish and poultry and good fats from sources such as extra virgin olive oil (11), present better metabolic control.

Given the high incidence of T1D in the Canary Islands (12, 13), we wanted to evaluate the influence of nonpharmacological factors such as adherence to the Mediterranean diet, body composition, physical activity, and mode of insulin delivery on HbA1c in our patients with T1D.

## 2 Methods

### 2.1 Design and population

This was an observational, cross-sectional study. Inclusion criteria: patients under 16 years of age living in Gran Canaria diagnosed with T1D according to ADA criteria (4), disease duration of more than 1 year, who were being followed by the Pediatric Endocrinology Unit of the Insular-Materno Infantil University Hospital (CHUIMI). All patients (and their parents) consented to participate in the study. The exclusion criteria included other types of diabetes, a diagnosis of T1D in the 12 months prior to the beginning of the study and a lack of consent to participate in the study.

### 2.2 Data collection

Patients from our unit were randomized (a randomized list including 280 eligible patients who met the inclusion criteria was created) before being invited to participate in the study, and those who accepted the study were scheduled for an appointment at the Pediatric Endocrinology Unit of CHUIMI. Only the first 200 patients were called due to time restrictions in order to perform the study. One phone call was attempted for each patient. At the scheduled appointment, patients’ height, weight, and body composition were measured and recorded. Patients were weighed and measured without shoes and with light clothing. Overweight was defined as a weight-for-height greater than 1 standard deviation above the median, and obesity was defined as a weight-for-height greater than 2 standard deviations above the median, as established in the World Health Organization (WHO) Child Growth Standards for Children >5 years of age (14). Body composition was measured using bioelectrical impedance analysis. A portable DC 360-S bioelectrical impedance analyzer (TANITA, Tokyo, Japan) was used to determine weight and estimate the percentages of fat and muscle for each child. Other recorded variables were age, disease duration, and method of insulin delivery.

### 2.3 Questionnaires

We used the Krece Plus short physical activity questionnaire to evaluate the amount of time dedicated to sedentary activities (score 1–5) and the amount of time dedicated to extracurricular physical activity (score 0–4) the patients or their parents thought they engaged in. The patients´ level of activity was classified as good, fair, or poor based on the resulting scores (0–3: bad; 4–6: fair; ≥ 7: good). We used the KIDMED Quick Nutrition Test to evaluate adherence to the Mediterranean diet. Both the Krece plus and KIDMED (15) questionnaires were obtained from the enKid study (16) and have been extensively used in Spain (11), Italy (17), Portugal (18), and other Mediterranean countries (19). The KIMED is a 16-item questionnaire that evaluates the adoption of healthy nutritional Mediterranean habits and scores +1 or − 1 depending on the answers (possible results ranging from −4 to 12). Interpretation of the final scores determined the presence of low, medium or optimal adherence to the Mediterranean diet (≤ 3: low-quality diet; 4–7: needs to be improved to adjust to the Mediterranean model; ≥ 8: optimal Mediterranean diet). Both questionnaires were answered by the children, when possible, with varying support from the parents depending on the children’s age.

### 2.4 Accelerometry data

We used the GENEActiv accelerometer (Activinsights Ltd., Kimbolton, United Kingdom) to evaluate the actual physical activity performed by our patients. The participants were instructed to wear the accelerometer on their dominant wrist for 5 consecutive days and nights. The data were recorded with an activity sample frequency of 100 Hz. The data were segmented into several time intervals labeled as six different classes (sedentary, light, moderate and intense physical activity, sleep and nonwear) using RStudio [RStudio Team (2022). RStudio: Integrated Development Environment for R (version 2022.07.2 + 576) (computer software). Boston, MA: RStudio, PBC] software and the data processing codes provided by the manufacturer. In this study, only data relating to moderate or intense physical activity were considered.

### 2.5 Metabolic control, insulin treatment, and technology adoption

The data on HbA1c levels, time in range (70–180 mg/dL; TIR), time above target, time below target, and coefficient of variation were extracted from the participants’ medical records. The average value from the last two visits was used. Good metabolic control was defined as an HbA1c level less than 7% (53 mmol/mol) or a percentage of time in the glucose range of 70–180 mg/dL above 70%. For all our patients receiving multiple doses of insulin (MDI), either a Dexcom G6 real-time continuous glucose monitor (CGM) or a Freestyle Libre 2 intermittently scanned CGM was used. Continuous subcutaneous insulin infusion (CSII) was performed via the hybrid closed loop system Medtronic 780G paired with Guardian 4 CGM and Smart Guard Technology or Tandem (t:slim X2) paired with Dexcom G6 CGM and Control-IQ Technology (all patients on CSII used hybrid closed loop systems).

### 2.6 Statistical analysis

SPSS version 21 (IBM SPSS Statistics for Windows, Armonk, NY, United States) and RStudio [RStudio Team (2022) were used. RStudio: Integrated Development Environment for R (version 2022.07.2 + 576; computer software). Boston, MA: RStudio, PBC] were used for statistical analysis of the data. For descriptive statistics, the mean and standard deviation were determined for normally distributed quantitative variables, while the median and interquartile range were calculated for nonnormally distributed variables. The Kolmogorov–Smirnov test was used to verify the normality of the distribution. Qualitative variables are described as frequencies. The hypothesis test was used to compare proportions and verify the difference between proportions, and Student’s *t* test was used to analyze the differences between the means of two samples. The Mann–Whitney U test was used for nonparametric variables. Correlation analysis was performed to assess the relationships between the KIDMED questionnaire score and HbA1c and TIR scores, between the enKid questionnaire score and the accelerometry score and between BMI and body fat percentage. Stepwise linear regression analysis (backwards) was used to identify factors independently associated with metabolic control (both HbA1c and TIR). A stepwise linear regression analysis was also used to evaluate which items included in the KIDMED questionnaire had an impact on HbA1c. *p* < 0.05 was considered to indicate statistical significance.

## 3 Results

**Figure 1:**
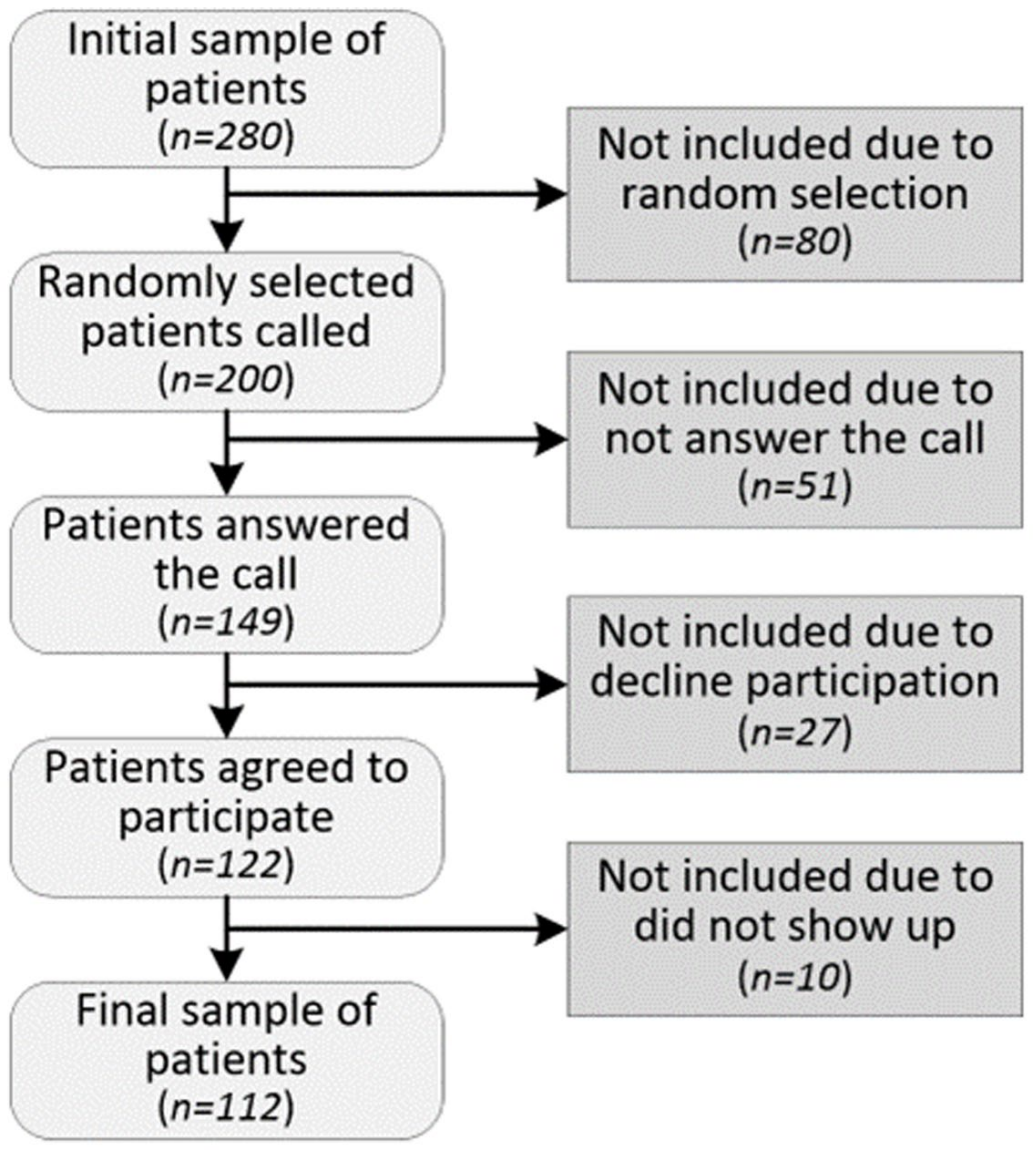
Recruitment process.

A total of 200 randomly selected T1D patients were initially contacted to participate in the study, and a total of 112 agreed to participate (56%). Accelerometry data were successfully recorded and processed for only 94 children. The recruitment process is summarized in Figure 1.

Table 1 describes the characteristics of the participants included in the study. There was a slight majority of MDI users compared to CSII users. The data showed significantly better HbA1c levels in patients treated with insulin pumps than in those receiving MDI [6.6% (49 mmol/mol) vs. 7.1% (54 mmol/mol); *p* value = 0.008].

**Table 1:** Characteristics of the participants included in the study.

	*N*/%	Descriptive variables
Sex (% Female)	58/51.8	
Insulin delivery systems (%hybrid closed loop)	49/45	
Average HbA1c [mean (CSII/MDI)]	111	6.93 (0.9) (6.6/7.1)
HbA1c < 7% (53 mmol/mol) (%)	67	60
HbA1c ≥ 7% & < 7.5% (≥ 53 & <58 mmol/mol) (%)	18	16.2
HbA1c > 7.5% (≥ 58 mmol/mol) (%)	26	23.4
Age (years. Median)	112	12 (5)
Time from onset (years. Median)	112	4 (4)
BMI (kg/m^2^/Zscore. Median)	112	19.7 (5.3)/ 0.19 (1.3)
Body fat percentage (mean)	111	22.65 (8.8)
Average daily screen time in hours (median)	111	2 (2)
Average enKid score (median)	112	5 (2)
Average KIDMED score (median)	112	8 (2)
Time in range (median)	110	73.5 (27.5)
Time in hypoglycemia (median)	108	3.3 (3.6)
Time in hyperglycemia (median)	103	25.25 (28.4)
Variation coefficient (median)	106	35.09 (6.8)
Average daily sleep hours (median)	92	6.45 (1.3)
Average daily moderate-intense activity hours (median)	91	3.3 (1.6)

Descriptive variables: mean (SD) or median (interquartile range); BMI, Body mass index.

**Figure 2:**
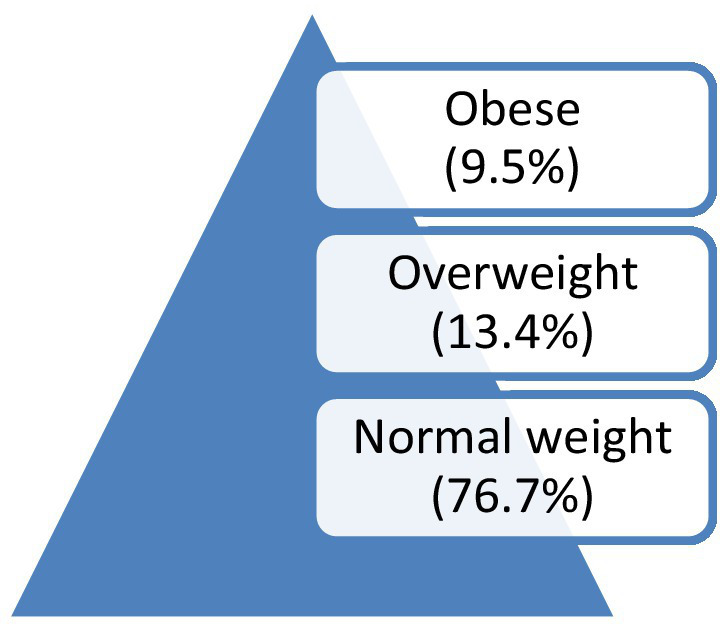
Weight distribution among patients with T1D.

In the study population, 76.7% of our patients had a normal weight, whereas 13.4 and 9.5% were overweight and obese, respectively (Figure 2).

To compare the results with those from Dominguez et al. (11), we performed a correlation analysis between HbA1c and the KIDMED score and obtained a negative correlation (*R* = −0.23; *p* = 0.016). A positive correlation was obtained between the TIR and KIDMED scores (*R* = 0.23; *p* = 0.014).

Correlation analysis revealed a moderate correlation between the enKid score and the average daily moderate-intense activity hours measured by accelerometry [*R* = 0.38; *p* = 0.000 (*N* = 91)]. Additionally, BMI and body fat percentage were strongly correlated [*R* = 0.7; *p* = 0.000 (*n* = 111)]. Table 2 summarizes the results of the correlation analyses.

**Table 2:** Summary of correlation analyses.

Items analyzed	*R*	*p* value
HbA1c/KIDMED score	−0.23	0.016
TIR/KIDMED score	0.23	0.014
enKID/Accelerometry	0.38	0.00
BMI/Body fat %	0.7	0.00

Backward stepwise linear regression analysis revealed that the variables significantly associated with HbA1c were the average score on the KIDMED questionnaire, insulin delivery method, years with T1D and age (Table 3, Model 1; other variables included in the analysis were BMI, body fat percentage, enKid score, sex, daily moderate-intense activity hours, and daily sleep hours). The model predicted 20% of the change in HbA1c. Body composition and activity measured by accelerometry or questionnaires were not significantly associated with HbA1c according to multivariate analyses.

**Table 3:** Models reflecting stepwise linear regression influencing metabolic control.

Model 1	Beta	*p* value	*R*	*R* ^2^	*p* value
(Constant)		0.000	0.453	0.205	0.036
Insulin delivery method	0.270	0.008			
Years with T1D	0.295	0.006			
Age (years)	−0.291	0.009			
Total KIDMED	−0.216	0.036			
Model 2					
(Constant)		0.000			
Insulin delivery method	−0.465	0.000	0.525	0.276	0.007
Years with T1D	−0.253	0.007			

Model 1: Step wise linear regression showing factors influencing HbA1c levels. Model 2: Step wise linear regression showing factors influencing time in range. The insulin delivery method used was multiple insulin injections. T1D, Type 1 diabetes.

Additionally, stepwise linear regression analysis showed that when analyzed independently, the only item included in the KIDMED questionnaire associated with HbA1c was not having eaten breakfast (beta = 0.32; *p* = 0.001).

A similar analysis was performed to determine the factors influencing the percentage of patients with a glucose concentration ranging from 70 to 180 mg/dL according to the sensor data. Insulin delivery method and diabetes duration were the only significantly associated variables, and the resulting model explained 27% of the variability in time in range (*p* = 0.007; Table 3, Model 2).

## 4 Discussion

This report evaluated the associations of body composition and nonpharmacological interventions (physical activity, insulin delivery methods, and adherence to a Mediterranean diet) with metabolic control. We showed a favorable association between self-reported nutritional habits, age, and the use of insulin pumps as well as a negative impact of the number of years with T1D on HbA1c levels.

The population studied included 45% of hybrid closed-loop pump users. With an average HbA1c of 6.9% (52 mmol/mol), our values are lower than those reported in Spanish (20) (7.3%) and international registries such as German-Austrian (DPV) and the American T1D exchange (21) [7.8% (62 mmol/mol) and 8.5% (69 mmol/mol), respectively]. The higher CSII adoption (45 vs. 25%) and the use of hybrid closed loop infusion systems in all of our CSII patients might help explain our lower values compared with those published in 2017 by Rica et al. (20). Compared to international registries, our CSII adoption rate was lower than that published by the DPV (89%) and the T1D exchange (65%). However, the fact that most of their patients did not use integrated systems at the time of the study might account for part of the difference (22, 23).

The KIDMED questionnaire has been used previously to assess adherence to the Mediterranean diet in pediatric T1D patients (11, 17). In our population, the median KIDMED score was 8, with 59% of our patients following an “optimal Mediterranean diet” (score ≥ 8). These results are very similar to those reported by Dominguez et al. (11) and Rebollo et al. (24) for a pediatric population living in southern Spain, both regarding the quality of the diet and the correlation of the latter with HbA1c and time in the glucose target range. Antoniotti et al. reported a similar study of a pediatric population in northern Italy (17). They reported a lower KIDMED score (median of 6, with only 29% of patients presenting a score ≥ 8) and did not find a significant correlation between the KIDMED score and metabolic control, only with the consumption of sweets and fish. In our case, the only independently associated component of the KIDMED questionnaire was “not having breakfast.” The difference in nutritional habits between the two populations might explain the difference in results. Levran et al. (25) recently reported an intervention targeting the quality of the diet in adolescents diagnosed with T1D in Israel in an attempt to improve the intake of these patients to better simulate the Mediterranean diet. They reported a significant improvement in metabolic control (TIR), as well as in other nutritional indicators, 6 months after the initiation of the intervention (25).

Regarding self-reported physical activity, the median enKid score was 5 in our sample, with only 35% of patients presenting an optimal score ≥ 7. On the other hand, the median number of hours of daily moderate-intense physical activity measured by accelerometry was 3.3 h (above the recommended 60 min/day). This difference is not surprising since the correlation between the enKid questionnaire score and activity measured with accelerometry was low, in agreement with the findings of other authors (26). A possible explanation could be that they probably measure different aspects of physical activity, and some authors recommend the complementary use of both strategies (27). With respect to the number of hours of data collected via accelerometry, we were surprised to find that, in our study, there were no significant associations between physical activity (measured by either accelerometry or the enKid questionnaire) and HbA1c. Nevertheless, our study is not the only one to do so. Shorey et al. (28) performed a meta-analysis to evaluate the impact of physical activity on metabolic control and reported a lack of effect on HbA1c. Watson et al. (29) reported a paradoxical relationship between physical activity and HbA1c, with increased physical activity relating to higher HbA1c values. The lack of significance in our study could be due to the small sample size, which was not large enough to detect a significant association. Additionally, there might have been confounding variables that were not considered. Third, the study may have been limited by the use of questionnaires and accelerometers, which can be subject to bias and inaccuracies. Additionally, simply, the relationship might not exist. On the other hand, other authors have reported a potential protective effect of exercise on β-cell health (30), as well as decreased HbA1c values in active children (10, 31) and adults (32) with T1D, with lower glucose values on active days, and through all type of structured exercises (aerobic, interval or resistance training). Apart from its debatable impact on metabolic control, exercise is highly recommended due to its positive effects on cardiovascular and overall health (33).

The effects of age and years with T1D on metabolic control have been widely studied. HbA1c values typically increase as puberty approaches and remain high until the beginning of the second decade for most patients with T1D (34, 35). Our results were slightly puzzling in the sense that the number of years with T1D was associated with higher HbA1c values, but age was inversely correlated with metabolic control. This difference was not explained by CSII use since further analysis revealed greater pump use in younger children. We think that the positive effect of age, along with the negative effect of the number of years with T1D, might be explained by selection bias, with older children with recent-onset T1D having greater participation.

A similar effect for time in range was found for the number of years after T1D and insulin delivery method. No relationship was found with age, hours of sleep, physical activity, or adherence to the Mediterranean diet.

Some of the strengths of our study are the randomization of patient selection (although self-selection cannot be ruled out), the number of patients studied and the simultaneous use of accelerometry and questionnaires to evaluate physical activity.

Some of the limitations of our study include the following: cross-sectional, retrospective study; possible self-selection, possibly with higher adherence to treatment and recommendations; and more hybrid closed-loop system users than our general population with T1D (45 vs. 35% of our total T1D pediatric population). Additionally, although the studied sample constitutes more than one-third of our total T1D population, the number of subjects might limit our ability to reflect all factors influencing metabolic control in our population.

In summary, our study evaluated the impact of body composition and nonpharmacological treatments on the metabolic control of our T1D population and revealed the significant effects of insulin delivery modality, adherence to the Mediterranean diet, age and years with T1D on HbA1c values. Body composition and physical activity, as measured by accelerometry or questionnaires (enKid), were not associated with HbA1c values. Our results highlight the need for strategies to improve metabolic control in pediatric patients approaching puberty. These findings contribute to our understanding of the factors influencing glycemic control in pediatric patients. However, further research including a larger number of subjects in our study and other populations is needed to validate and expand upon these findings.

## Data availability statement

The original contributions presented in the study are included in the article/supplementary material, further inquiries can be directed to the YN-M, yeraynm@hotmail.com.

## Ethics statement

The studies involving humans were approved by Ethics Committee of Las Palmas University Hospital Dr. Negrín. The studies were conducted in accordance with the local legislation and institutional requirements. Written informed consent for participation in this study was provided by the participants’ legal guardians/next of kin. Written informed consent was obtained from the minor(s)’ legal guardian/next of kin for the publication of any potentially identifiable images or data included in this article.

## Author contributions

YN-M: Conceptualization, Data curation, Formal analysis, Funding acquisition, Investigation, Methodology, Project administration, Resources, Supervision, Writing – original draft, Writing – review & editing. AP-L: Investigation, Resources, Writing – review & editing. NS-R: Investigation, Resources, Writing – review & editing. MB-B: Investigation, Resources, Writing – review & editing. HF: Data curation, Formal analysis, Investigation, Resources, Software, Writing – review & editing. SL-L: Data curation, Writing – review & editing. SQ: Data curation, Writing – review & editing. AD: Data curation, Writing – review & editing. ML: Data curation, Writing – review & editing. MG: Data curation, Writing – review & editing. EC: Data curation, Writing – review & editing. AW: Conceptualization, Data curation, Formal analysis, Investigation, Supervision, Writing – review & editing.
